# Longitudinal fiber dysfunction assessed during cine-cardiac magnetic resonance imaging is an independent predictor of adverse cardiac events

**DOI:** 10.1186/1532-429X-18-S1-P135

**Published:** 2016-01-27

**Authors:** Satish J Chacko, Vibhav Rangarajan, Nikhil Jariwala, Simone Romano, Jaehoon Chung, Afshin Farzaneh-Far

**Affiliations:** 1 grid.185648.60000000121750319Cardiology, University of Illinois at Chicago, Chicago, IL USA; 2 grid.5611.30000000417631124Medicine, University of Verona, Verona, Italy; 3 grid.26009.3d0000000419367961Cardiology, Duke University, Durham, NC USA

## Background

Left ventricular systole involves coordinated contraction of longitudinal, circumferential, and radial myocardial fibers. Longitudinal fiber dysfunction appears to be an early marker for a number of pathological states. We hypothesized that reduced mitral annular plane systolic excursion (MAPSE) measured during cine-Cardiac Magnetic Resonance (CMR) imaging reflects changes in longitudinal fiber function and may be an early marker for adverse cardiovascular outcomes.

## Methods

400 consecutive patients with known or suspected coronary artery disease undergoing CMR were prospectively enrolled. Lateral MAPSE was measured in the 4-chamber cine view by two independent observers. Patients were prospectively followed for major adverse cardiac events (MACE) - death, non-fatal myocardial infarction, hospitalization for heart failure or chest pain, and late revascularization. Cox proportional hazards regression modeling was used to identify factors independently associated with MACE.

## Results

**Figure 1:**
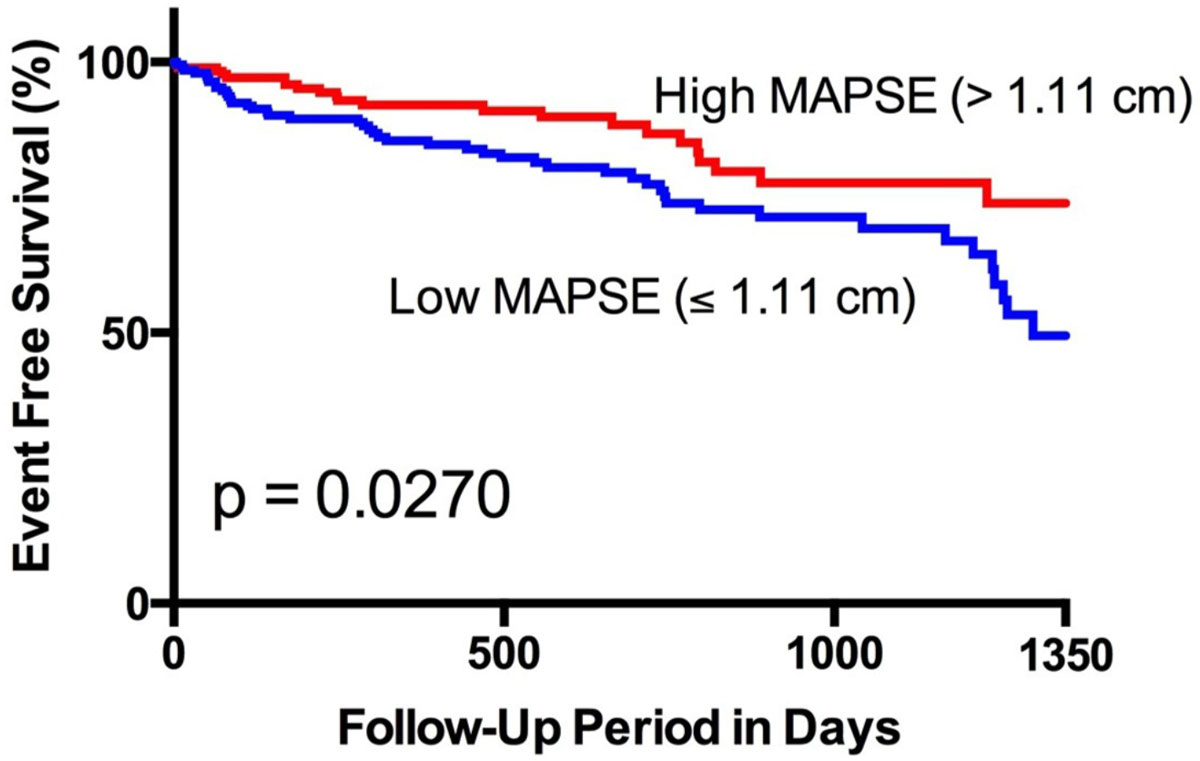
**Kaplan-Meier curves for MACE in patients with MAPSE above and below the median**.

**Figure 2:**
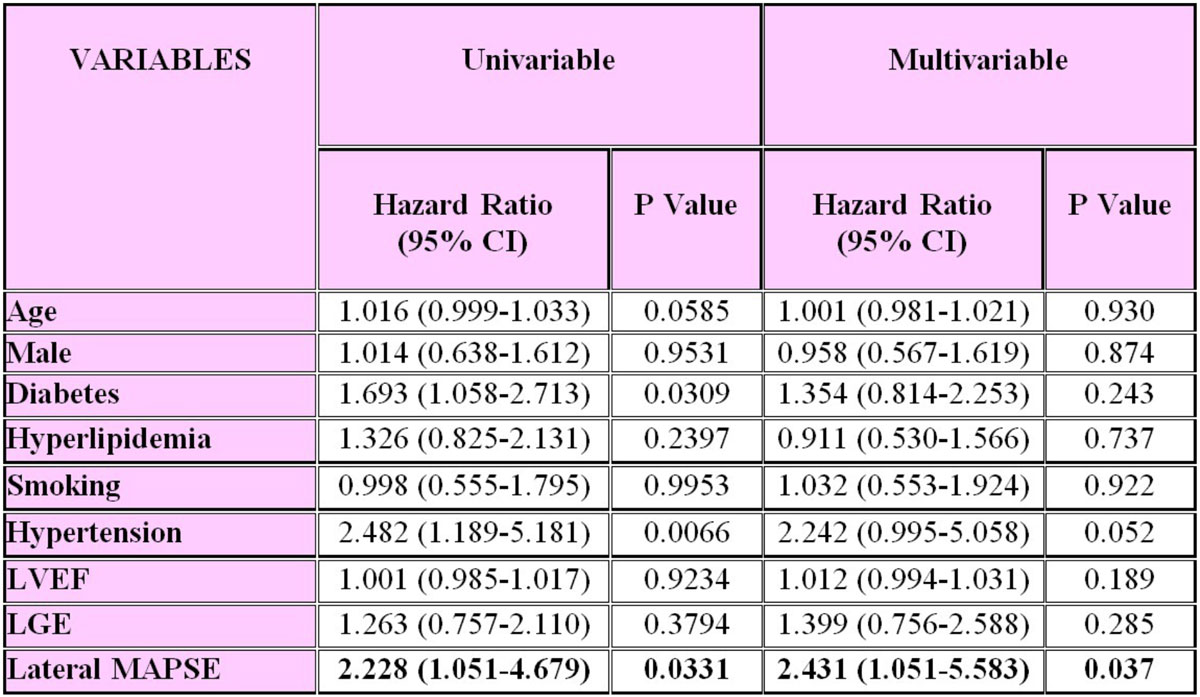
**Univariable and multivariable predictors of MACE**.

The mean age of the study population was 58(± 15) years, with a mean ejection fraction of 59(± 14%). 31% of the individuals had known coronary artery disease and 33% were diabetic. 72 MACE occurred during a median follow-up of 14.5 months. By Kaplan-Meier analysis, patients with lateral MAPSE ≤1.11 cm (median) experienced significantly higher incidence of MACE than patients with a MAPSE >1.11 cm (p = 0.0270) (Figure 1). After adjustment for established predictors (ejection fraction, age, sex, diabetes, hyperlipidemia, smoking, hypertension, late gadolinium enhancement) lateral MAPSE remained a significant independent predictor of MACE (HR = 2.43 per cm decrease; p = 0.037) (Figure 2).

## Conclusions

Longitudinal fiber dysfunction assessed with lateral MAPSE during cine-CMR is an independent predictor of MACE in patients with known or suspected coronary artery disease.

